# The association of combat exposure and injuries with behavioral health problems among enlisted female Army members returning from Iraq or Afghanistan

**DOI:** 10.1186/1940-0640-10-S1-A1

**Published:** 2015-02-20

**Authors:** Rachel Sayko Adams, Ruslan V Nikitin, Nikki R Wooten, Thomas V Williams, Mary Jo Larson

**Affiliations:** 1 Shneider Institutes for Health Policy, Heller School, Brandeis University, Waltham, MA, 02453, USA; 2 College of Social Work, University of South Carolina, Columbia, SC, 29208, USA; 3 Defense Health Agency/Department of Defense, Falls Church, VA, 22042, USA

## Background

The association between combat exposure and injuries with postdeployment behavioral health problems has been well studied, yet few studies examine separately the exposures and problems reported by females. This study explores the postdeployment self-report of combat exposure and injury and the postdeployment behavioral health screening results of Army women.

## Materials and methods

Using longitudinal data from The Substance Use and Psychological Injury Combat Study, we selected a sample of enlisted female Army members returning from Afghanistan or Iraq in FY2008–2011, who completed a health questionnaire within 60 days of their deployment end date (N = 42397; 6.6% of cohort). Combat score was constructed as an ordinal variable (0–3) based on four items: being wounded, injured, assaulted, or hurt; encountering dead bodies/seeing people killed; firing a weapon; and being in danger of being killed. Multivariate logistic regression models examined how combat score, demographic, and deployment characteristics predicted posttraumatic stress disorder (PTSD), depression, and at-risk drinking. Models were stratified by component: Active Duty (AD) and National Guard/Reserves (NG/Rs).

## Results

A substantial number of females reported being injured, wounded, assaulted, or hurt (17% of AD and 29% of NG/Rs). Over 14 percent reported encountering dead bodies/seeing people killed, 1 percent fired a weapon, and 19 percent were in danger of being killed. Approximately 12 percent had a combat score of 2+. In all models, female members with combat exposure had increased odds of behavioral problems. There was a dose-response relationship, such that as combat score increased, the odds of each behavioral problem increased. The risk was most striking for PTSD. Among AD females, those with a combat score of 1 had 4.4 times the odds of having PTSD compared to those with a combat score of 0 (95% confidence interval: 3.82–4.98). The odds increased to 20.6 for those with a combat score of 3. Results were similar for NG/R women.

## Conclusions

Combat injury while deployed may be greater than expected among enlisted female Army members, particularly among NG/Rs. Even those with a score of 1 on a four-item combat scale had a significant increase in odds of PTSD. A high proportion of females who deploy to combat zones may benefit from early intervention to promote postdeployment health and reduce long-term behavioral health problems.

